# Radiology and A Radiologist: A Keystone in the Turmoil of Trauma Setting

**DOI:** 10.7759/cureus.14267

**Published:** 2021-04-02

**Authors:** Khadija Hussain, Deepak Verma, Amena Firoz, Karez S Namiq, Maham Raza, Muhammad Haris, Manel Bouchama, Safeera Khan

**Affiliations:** 1 Radiology, California Institute of Behavioral Neurosciences & Psychology, Fairfield, USA; 2 Internal Medicine/Family Medicine, California Institute of Behavioral Neurosciences & Psychology, Fairfield, USA; 3 Pediatrics, California Institute of Behavioral Neurosciences & Psychology, Fairfield, USA; 4 Oncology, California Institute of Behavioral Neurosciences & Psychology, Fairfield, USA; 5 Internal Medicine, California Institute of Behavioral Neurosciences & Psychology, Fairfield, USA; 6 Internal Medicine, Royal Lancaster Infirmary, Health Education England North West, Lancaster, GBR

**Keywords:** imaging, trauma, imaging and trauma, essentials of trauma imaging

## Abstract

Traumatic injuries are one of the leading causes of morbidity and mortality. Precise diagnosis and management in the golden hour are key to decrease morbidity and mortality. History and physical examination alone are insufficient to avoid misdiagnosis. In this article, we tried to determine the role of a radiologist and an appropriate imaging modality in a trauma setting.

We conducted a literature review of published research articles. We used the keywords imaging, trauma, imaging and trauma, and trauma imaging essentials were used on PubMed and Google Scholar. The articles published in the English language from 2015 to 2020 with full free text available were included. Using the medical subject heading (MeSH) strategy, "diagnostic imaging" (Major {Majr}) and "multiple trauma/diagnostic imaging" (Mesh) on PubMed, we identified 34 papers after applying the inclusion and exclusion criteria. Twenty articles were finally selected which included studies from 2015 to 2020 with articles focusing on the adult population and acute cases.

A radiologist and imaging modalities are the essential parts of a trauma setting to lower morbidity and mortality. X-rays and Extended Focussed Assessment with Sonography for Trauma (eFAST) are the first-line imaging modality in the acute trauma setting. However, the CT scan is the most sensitive modality that should be done to avoid misdiagnosis depending upon the patient's history and physical examination.

## Introduction and background

According to World Health Organization (WHO), there are around 14,000 deaths per day due to traumatic injury worldwide, i.e., one death every six seconds. Among traumatic injuries, road traffic accidents are the leading cause. Other causes of injuries include falls, drowning, burns, sports injuries, acts of violence, and wars [1]. The key to decrease mortality and lifelong disability following trauma is early diagnosis. However, mere history and physical examination often have low sensitivity and specificity for accurate diagnosis of acute traumatic injuries.

Around 20-50% of patients with blunt polytrauma are misdiagnosed without radiological imaging [2]. For prompt, accurate diagnosis and management, the most appropriate imaging modality is integral [3]. With the advancement of imaging modalities, it is critical to procure knowledge as "The eye can't see what the mind is not prepared to comprehend" [4]. Advanced trauma life support (ATLS) has been a part of trauma protocol in more than 60 countries. It also includes trauma series (plain X-ray of the cervical spine, chest, and pelvis), a focused assessment with sonography for trauma (FAST) examination, and a CT scan indicating the importance of radiology in "golden hour". Trauma series and FAST are included in the primary survey of ATLS. Both trauma series and FAST can be very useful in unstable polytrauma patients as both can be done in the trauma room without interruption in resuscitation [2].

Since 1980, FAST has been used to detect free fluid in abdominal trauma patients as a first-line modality. Free fluid is detected in perihepatic space and Morrisson's pouch, perisplenic space, the pouch of Douglas, and pericardial space. Recently, the right pleural space, the left pleural space, and two anterior pleural spaces have also been added in FAST, which modified the term into eFAST (extended FAST) [5-7]. Non-contrast CT is the first-line imaging modality for those patients with a head injury who present in an emergency with a neurological deficit [8]. Sir Hounsfield first introduced a CT scan in 1972 that was X ray-based single detector scanner. Now, with recent advancements, CT scanners with more spatial resolution have evolved, which takes just a few seconds to image the whole body [2].

However, despite the advancements in radiology, there is still no unanimous decision as to which imaging modality is best for which type of trauma and body part to avoid misdiagnosis. Other areas that need further research include "can a radiologist change clinician's perspective of thinking?" "How radiological errors can affect a patient's fate?" "Is there a necessity for more coordination between clinician and radiologist to avoid unnecessary radiation exposure?"

In this review article, we tried to determine which imaging modality is best for which type of trauma and body part and what essential role a radiologist can play in a trauma setting to change a patient's outcome.

## Review

We conducted a literature review of published research articles to determine the role of radiology in the trauma setting.

Methods

We searched the articles published in the English language from 2015 to 2020 with full-text on PubMed and Google Scholar. Keywords used on PubMed included imaging, trauma, imaging and trauma, and essentials of trauma imaging. Using the keywords "imaging" showed 1,88,727 results; "trauma" showed 61,393 results; "trauma and imaging" showed 14,163 results; and "essentials of trauma imaging" showed 285 results. Out of these results, 22 articles were selected that seemed to be relevant after reviewing the abstract. By using the medical subject heading (MeSH) search strategy, keywords, "diagnostic imaging" (Majr) and "multiple trauma/diagnostic imaging" (Mesh) were built and then searched on PubMed using the same filters as mentioned above. It showed 34 results. Out of these 34 results, three articles were selected after reviewing the abstract. Duplicates papers were removed and inclusion and exclusion criteria were applied. After the further screening of the identified articles in detail, 20 research papers were finally chosen to be reviewed.

Inclusion and Exclusion Criteria

We chose the articles that were published in the last five years from 2015 to 2020 and those focused on the adult population. The articles focusing only on acute trauma cases were included. The articles mainly discussing radiological diagnostic imaging techniques and that were relevant to the research question were finalized for the review.

We did not include any research papers that focused on the pediatric population. Gray literature and unpublished research were also not included in our review. Papers that had only abstracts available were also excluded.

Results

Out of these 20 selected studies, six of them discussed the role of ultrasonography. Nine were related to CT scans. One study was about the combined role of CT and MRI. Two studies examined polytrauma imaging. The remaining two were about errors in a trauma setting and imaging in limited resources. Table 1 shows all selected studies.

**Table 1:** Details of All Selected Studies eFAST = extended focused assessment with sonography for trauma; US = ultrasound; MDCT = multidetector computerized tomography; USG = ultrasonography

Study	Year of Publication	Design	Conclusion
Imaging protocols for trauma patients: trauma series, extended focused assessment with sonography for trauma, and selective and whole-body computed tomography [2]	2016	Review article	To provide immediate and critical care to the patients, whole-body imaging has evolved as an important tool.
Dual-phase CT for the assessment of acute vascular injuries in high-energy blunt trauma: the imaging findings and management implications [3]	2016	Review article	Dual-phase CT is highly useful to detect vascular injuries and plays an important role to change a patient’s outcome.
Abdominal and pelvic trauma: misses and misinterpretations at multidetector CT: trauma/emergency radiology [4]	2017	Review article	Most commonly misdiagnosed injuries include abdominal, mesenteric, and vascular injuries. CT is the investigation of choice.
The effect of extended‑focused assessment with sonography in trauma results on clinical judgment accuracy of the physicians managing patients with blunt thoracoabdominal trauma [5]	2019	Cross-sectional study	eFAST plays an important role to enhance the sensitivity of history and physical examination.
Trauma (FAST) in 2017: what radiologists can learn [6]	2017	Review article	FAST has high sensitivity and specificity for intraabdominal free fluid but low sensitivity for solid organ damage.
Focused assessment with sonography for trauma: current perspectives [7]	2017	Review article	FAST plays a key role to triage patients in trauma settings.
Skull base-related lesions at routine head CT from the emergency department: pearls, pitfalls, and lessons learned [8]	2019	Review article	History and the clinical symptoms are intriguing points for a radiologist to alter a patient’s outcome.
Emergency thoracic US: the essentials [9]	2016	Review article	The US is a very useful modality for acute thoracic injuries.
Diagnostic value of abdominal follow-up sonography in polytrauma patients: a retrospective study [10]	2020	Retrospective cohort	Abdominal follow-up sonography can be safely missed in the absence of findings on multi-slice CT.
Predictive value of focused assessment with sonography for trauma (FAST) for laparotomy in unstable polytrauma Egyptians patients [11]	2017	Cross-sectional study	FAST is an accurate examination for intraabdominal fluid but it has limitations.
Multidetector CT of mandibular fractures, reductions, and complications: a clinically relevant primer for the radiologist [12]	2016	Review article	A Radiologist’s knowledge is critical to help in the decision-making process of a reconstructive surgeon.
The role of emergency radiology in spinal trauma [13]	2016	Review article	MDCT detects bone injury while MRI detects soft tissue injuries.
MDCT imaging of traumatic brain injury [14]	2016	Review article	CT is the investigation of choice not only for detecting injury but also for the follow-up.
Pancreatic trauma: the role of computed tomography for guiding therapeutic approach [15]	2015	Retrospective cohort	CT scan not only probes the pancreatic injury but also helps in formulating a grading for management.
Multidetector CT of surgically proven blunt bowel and mesenteric injury [16]	2017	Review article	The knowledge of bowel and mesenteric injuries is essential for a radiologist to alter a patient’s outcome.
Can multidetector CT detect the site of gastrointestinal tract injury in trauma? A retrospective study [17]	2017	Retrospective cohort	CT scan can detect gastrointestinal injury but localization of the exact site of injury is difficult especially for a large bowl.
Gastric blunt traumatic injuries: a computed tomography grading classification [18]	2017	Retrospective cohort	A radiological grading by a radiologist is worthwhile not only for diagnosing but also for the management of patients.
Systematic review: effect of whole-body computed tomography on mortality in trauma patients [19]	2015	Review article	Whole-body CT diminishes the mortality rate regardless of vital stability.
Errors in imaging patients in the emergency setting [20]	2016	Review article	The key to decrease radiological errors are sound understanding, selection of accurate imaging modalities, and familiarity.
Imaging in trauma in limited-resource settings: a literature review [21]	2019	Review article and meta-analysis	History, physical examination, X-rays, and USG can play a role in the absence a of CT scan.

Discussion

Most of the difficult-to-diagnose life-threatening injuries included head and neck, thorax, and abdomen. To avoid misdiagnosis and for identifying them accurately, different imaging modalities are needed.

Role of X-rays and Ultrasonography

**Figure 1:**
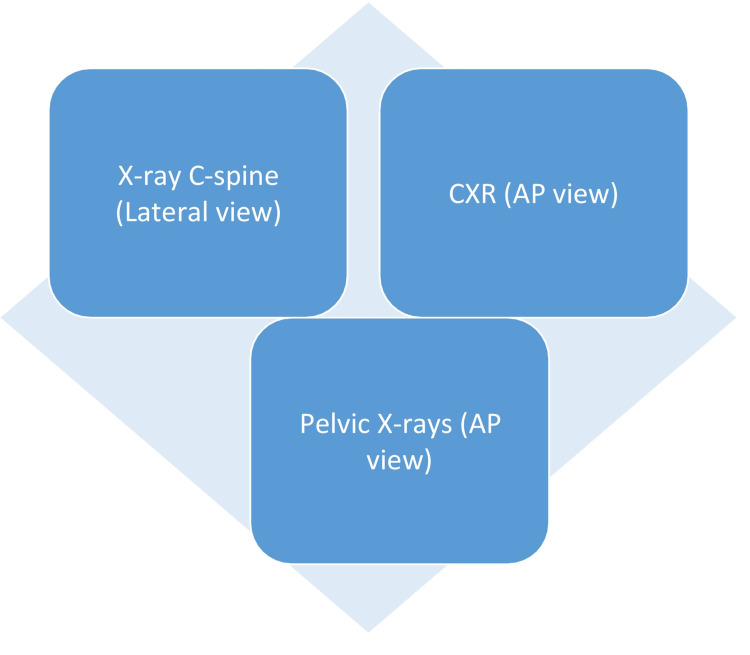
Trauma Series C-spine = cervical spine; CXR = chest X-ray; AP = anteroposterior

According to O׳Keeffe et al., the constituents of trauma series of radiographs are cervical spine X-ray (lateral), chest X-ray (anteroposterior view) in the supine position, and a pelvic X-ray (anteroposterior view) (Figure 1). However, the cervical spine X-ray is not mandatory as once the spine is immobilized, there is very little chance of worsening the injury [2]. So cervical spine radiograph can be delayed according to the patient's condition.

For thoracic injury, a chest X-ray (AP view) is helpful. It is taken in the supine position due to the uncertainty of a diagnosis of the cervical spine injury in an unstable patient. A portable chest X-ray can be useful in the early diagnosis of pneumothorax or hemothorax. Pelvic X-rays probe into any fracture linked with a major source of bleeding from vascular injuries. X-rays can be a useful tool to aid in the history and physical examination, but there are likelihoods of misdiagnosis due to its low sensitivity [2]. Therefore, in polytrauma patients, we can't simply rely on X-rays.

According to a study by Wongwaisayawan et al., ultrasonography (USG) is widely used, as it has numerous benefits like no radiation exposure, easy availability, noninvasive, and financially inexpensive. So, to rule out life-threatening thoracic injuries in an unstable patient, portable X-rays and eFAST are the mainstay imaging modalities. However, in a stable polytrauma patient, CT should be the preference. Although chest X-ray is specific for rib fractures, sensitivity is low specifically in a non-displaced fracture. USG is more sensitive than X-rays in such cases. Similarly, in pneumothorax, hemothorax, and pleural effusion, the sensitivity and specificity of USG are more than Chest X-rays [9].

A study by Bagheri-Hariri et al. revealed that the addition of the eFAST and physical examination increased the sensitivity to diagnose hemoperitoneum from 38.5% to 76.9%. While in the case of hemothorax, sensitivity increased from 20% to 80% [5]. eFAST has a higher sensitivity of 43-77% compared to chest X-rays with a sensitivity of 11-21%. However, an important aspect to contemplate is that if USG is performed too soon, there may be a plausibility of misdiagnosis. In such false-negative cases, repeated USG examinations might be needed [6]. However, according to a study by Lichtenstein et al., abdominal follow-up sonography (AFS) that is usually performed six hours after admission can be safely skipped if there is no finding on multi-slice CT scan [10].

Elbaih and Abu-Elela discussed the role of FAST for abdominal trauma. According to this study, all those cases with findings on the FAST scan ended up in an exploratory laparotomy. However, there was a 7.4% misdiagnosis (false negative). According to this study, the FAST examination cannot detect solid parenchymal damage, diaphragm injury, and retroperitoneal injuries [11]. FAST is a valuable means to triage the patients. However, if there is a finding on the FAST examination in a hemodynamically stable patient, CT is recommended to assess organ damage [7]. So a radiologist plays a role of a beacon in the darkness of multiple injuries.

Role of CT and MRI

CT scan is an advanced version of X-rays in which cross-sectional images of body tissues are taken using a sequence of X-ray images taken from multiple angles. At the same time, MRI uses magnetic fields to image the body organs. A study by Iacobellis et al. narrated that in the case of blunt chest or abdominal trauma, multidetector CT (MDCT) should be used to detect any suspected vascular injury as MDCT can affect management protocol from conservative management for a simple intimal tear to surgical intervention for vascular injuries. Moreover, detecting the actual vessel injured and finding out whether the arterial or venous type of vessel is injured affect the ultimate management. The arterial injury needs immediate intervention while venous injury can be managed initially conservatively. As in the case of aortic intramural hematoma (vessel wall thickens to 0.5mm in the absence of blood flow sign), any delay in the diagnosis can lead to aortic dissection. In this way, a radiologist plays a key role to avoid unnecessary intervention or preventing worse outcomes [3].

A study by Dreizin et al. depicted the role of the MDCT in a case of mandibular fracture. According to this study, a radiologist plays a key role in the decision-making process of a reconstructive surgeon as MDCT plays a vital role in the diagnosis and the management process [12]. Guarnieri et al. showed that the patients with a suspicion of bony spine injuries need MDCT while detecting soft tissue injuries, MRI is indicated [13].

Lolli et al. showed that CT is the investigation of choice not only for an acute head injury but also for subsequent follow-up. However, if the patient's condition deteriorates despite no abnormality found on CT, then MRI is recommended [14]. MRI is not widely used in trauma settings as it takes more time than a CT scan.

Abdominal and mesenteric injuries are a challenge for a radiologist as the delay in diagnosis or misinterpretation can cost a patient's life. According to Patlas et al. the spleen, liver, kidneys, small bowel, mesentery, colon, diaphragm, bladder, pancreas, and vascular injuries are the most commonly misdiagnosed. In hemodynamically stable cases of abdominal trauma, a CT is the investigation of choice, particularly to detect pancreatic injuries. According to a study by Panda et al. CT has a sensitivity of 96.7% for gastrointestinal tract injuries. The most commonly misdiagnosed part of the abdomen, even on CT, is the colon. Intraluminal air, extraluminal air, hypo enhancement, hyper enhancements, and wall discontinuity are the signs to detect and localize bowel injury. Furthermore, having sound knowledge of anatomy and common complications is critical for a radiologist to alter the patient's fate [4,15-17].

Solazzo et al. particularly focussed on gastric injuries. They formulated a grading score depending upon the radiological findings to triage the patients requiring surgical or conservative management. According to this study, CT is the gold standard investigation for abdominal injuries [18]. O׳Keeffe et al. proposed the use of whole-body CT (WBCT) instead of individualized organ CT. According to them, patients with spinal injuries, multiple rib fractures, penetrating abdominal injuries, electric burns of more than 20%, penetrating head, neck, and torso injuries, and fall from the height of more than ten feet need WBCT as it saves time for immediate diagnosis and emergency management [2]. According to a systematic review by Hajibandeh and Hajibandeh, WBCT lowers the mortality rate in blunt trauma patients regardless of hemodynamic stability [19].

Causes of Radiological Errors

Common causes of radiological errors include incomplete and inappropriate history, complex injuries, lack of knowledge of a radiologist, poor selection of imaging modality, and lack of communication between radiologists and clinicians due to urgency of rapid diagnosis and management. The most common errors include non-displaced fractures [20].

Imaging in Limited Resources

**Figure 2:**
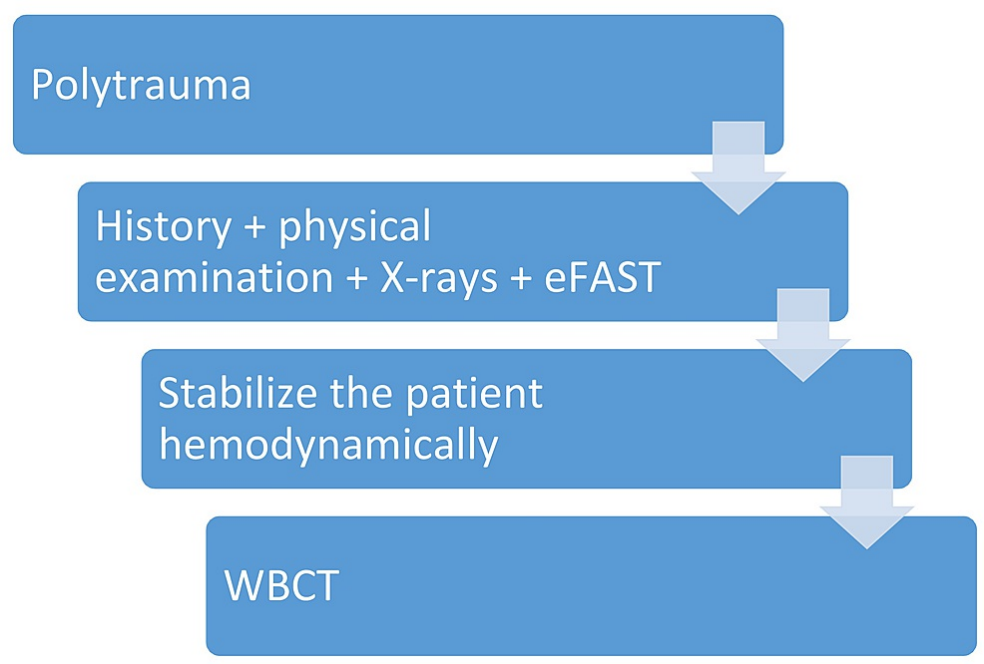
Trauma Imaging Algorithm eFAST = extended focused assessment with sonography for trauma; WBCT = whole body computerized tomography

Albeit USG and X-rays have less sensitivity, still, in the case of limited resources, both X-rays and USG have reaped a standpoint. Proper history, physical examination, low dose X-rays (LODOX), USG, laboratory investigations, and adequate monitoring can provide valuable information in the absence of a CT scan facility [21]. Figure 2 shows an algorithm based on the discussion.

Limitations

This study did not include the pregnant and pediatric population therefore cannot discuss the best imaging modalities in these population groups. Moreover, most of the studies that were included were either retrospective observational studies or review articles. More randomized controlled trials (RCT)s are needed to explore this topic as there are so many questions that need to be answered.

## Conclusions

This review article indicates the significance of using different imaging modalities in trauma settings that are more accessible to use and have a higher diagnostic sensitivity, thus decreasing post-traumatic mortality and morbidity. Although CT is the most sensitive modality in acute trauma, X-rays and eFAST are widely used in trauma settings due to the easy availability, low radiation exposure, the ability to perform at the patient's bedside. If the patient is hemodynamically unstable, then it is better to start with X-rays and USG to get a clue about further management. Still, in a hemodynamically stable patient, WBCT should be preferred according to the patient's history and physical examination to avoid misdiagnosis.

A trauma setting cannot be run without radiological imaging and a radiologist. Similarly, a radiologist needs proper history to reach a definitive diagnosis. Both clinician and a radiologist need to work together to achieve the best patient care. Moreover, a radiologist should recommend the best imaging modality according to a patient's condition if required. More RCTs are still required, including both clinicians and radiologists, to simplify the trauma algorithm further and decrease patient radiation exposure. The role of MRI in a trauma setting is still unexplained. As a CT scan exposes the patient to radiation so it is needed to find out whether we can use MRI in the trauma setting or can we somehow decrease its operating time to get a rapid diagnosis.
